# CEO Turnover, Leadership Identity, and TMT Creativity in a Cross-Cultural Context

**DOI:** 10.3389/fpsyg.2021.610526

**Published:** 2021-08-25

**Authors:** Pengfei Rong, Chao Wang

**Affiliations:** College of Philosophy, Law and Political Science, Shanghai Normal University, Shanghai, China

**Keywords:** CEO turnover, TMT creativity, leadership identity, moderating effect, cross-cultural context

## Abstract

Whether chief executive officer (CEO) turnover can improve top management team (TMT) creativity is an important issue that remains to be solved. Based on the theoretical background of CEO turnover, team creativity, and cross-cultural context, this study proposes a theoretical model to answer the question and introduces leadership identity as a moderator simultaneously. The multiple regression analysis of data obtained from 903 executives in 104 top management teams revealed CEO voluntary resignation/internal succession pattern, CEO voluntary resignation/external succession pattern, and CEO forced resignation/internal succession pattern separately had a significant positive impact on TMT creativity in a cross-cultural context; leadership identity partially moderated the relationship between CEO turnover and TMT creativity. According to these findings, only three patterns of CEO turnover could promote TMT creativity, and leadership identity enhanced the positive effects of CEO voluntary resignation/internal succession pattern, CEO voluntary resignation/external succession pattern, and CEO forced resignation/internal succession pattern on TMT creativity in a cross-cultural context. These made up for the lack of theoretical research on the relationships among CEO turnover, TMT creativity and leadership identity, which could provide the scientific guidance to conduct the CEO turnover practice and improve TMT creativity in a cross-cultural context.

## Introduction

With the integration of the world economy and the increasing number of global cities, more and more enterprises go abroad to carry out the transnational business activities and form the cross-cultural enterprises, thus cross-cultural management has become a hot research topic. The research about the cross-cultural management focuses on how to overcome the conflicts caused by the cultural differences and carry out the effective management in the cross-cultural context (Sun, 2008). Cultural conflict is a process of mutual opposition and exclusion between different forms of culture or cultural elements, and it not only refers to the conflicts that the multinational corporations are contradictory with different cultural concepts of the host country when they operate in the other countries but also includes the conflicts arising because employees belong to countries with different cultural backgrounds in an enterprise (Zhang, 2000). Especially for top management team (TMT) in a multinational corporation whose members come from different countries and have great differences not only in age, educational background, and work experience but also in ideas,beliefs, values, and intrinsic motivations, they are easier to produce the various contradictions and conflicts in the process of the collective decision-making (Segaro et al., 2014).

Top management team composed of CEO (chief executive officer), general manager, deputy general manager, and senior managers who report directly to the above is a small group of senior managers, which is led by the CEO, and undertakes the important mission of formulating and implementing strategic decisions as the core decision-making group of an enterprise (Rong et al., 2018). In a cross-cultural context, the formulation and implementation of strategic decisions are fraught with uncertainty and complexity, and these require TMT to be innovative; TMT members must be able to get the new ideas and methods to solve the complex decision-making problems creatively (Rong et al., 2019a). TMT creativity is the ability for TMT members to present the innovative and practical ideas for the management practice and strategic decisions (Shin and Zhou, 2007). In the past, scholars mainly studied team creativity in the general teams, revealed the effects of task difficulty, and team diversity on team creativity and the curvilinear relationship between ethical leadership and team creativity. For example, http://doi.or.kr/10.PSN/ADPER6803014815; Chae et al. (2015) found managers needed to increase team diversity so that their teams could maximize team creativity through rigorous exploration and exploitation in the case of a difficult task; Mo et al. (2019) revealed an inverted U-shaped relationship between ethical leadership and team creativity, and the teams would exhibit more creativity when there was a moderate level of ethical leadership than when there were very low or high levels. Compared with a general team, TMT in a multinational corporation is at a higher level of management, undertakes a larger mission, handles the more complex tasks, and requires team members to have more knowledge and skills; therefore, TMT creativity in a cross-cultural context is worthy of study by scholars. Fortunately, some scholars have noticed this and studied the internal mechanism, in which paternalistic leadership and transformational leadership influenced TMT creativity (Chang et al., 2016; Kim, 2017). These studies show team creativity is the crystallization of collective wisdoms to TMT members, and the leadership behavior is a crucial factor affecting TMT creativity; the scientific leadership behavior contributes to motivating TMT creativity. Therefore, if the CEO as the leader of TMT changed, how would it affect TMT creativity in a cross-cultural context?

The CEO employed by the board of directors is the highest executive officer who represents the board of directors to deal with the day-to-day affairs of the enterprise (You et al., 2020). CEO as the leader of TMT is the core maker of the strategic decision-making, his management level and decision-making ability often determine the rise and fall of an enterprise (Zhang et al., 2005), and the selection and the turnover of CEO are the most important things for the enterprise as a result (Sun and Huang, 2012). In the 1960s, Grusky (1960) first put forward the empirical research model of CEO turnover and pointed out CEO turnover was the key factor that affected enterprise structure and leads to enterprise instability. Since then, scholars have mainly studied the internal mechanism of CEO turnover (Huang et al., 2019) and the relationship between CEO turnover and corporate value based on the principal-agent theory (Chen et al., 2019; Jarva et al., 2019). CEO turnover in a multinational corporation includes two successive events: the former CEO resignation and the successor CEO succession; it is a complex process and may lead to the change of top managers and enterprise value in different degrees (Zhang et al., 2005), and results in the reorganization of TMT and the changes of both TMT creativity and enterprise strategies, but, up to now, no scholars have studied its internal mechanism. In addition, social identity theory holds that the learning and imitation of subordinates toward the leaders are based on a high degree of recognition of the leaders (Ambrose et al., 2018). Therefore, the learning and the imitation coming from TMT members to CEO are premised on the recognition toward CEO and the acceptance of his working concept. Leadership identity as a cognitive state that reflects the appreciation and acceptance of TMT members toward CEO (Ambrose et al., 2018; Li et al., 2018) is an important moderating factor that affects the relationship between CEO turnover and TMT creativity in a cross-cultural context, but how to adjust is still unknown. Therefore, can CEO turnover improve TMT creativity in a cross-cultural context? And how can the leadership identity of TMT members play a moderating role in this process? This study will try to answer these questions.

## Theoretical Background

### CEO Turnover

**Figure 1:**
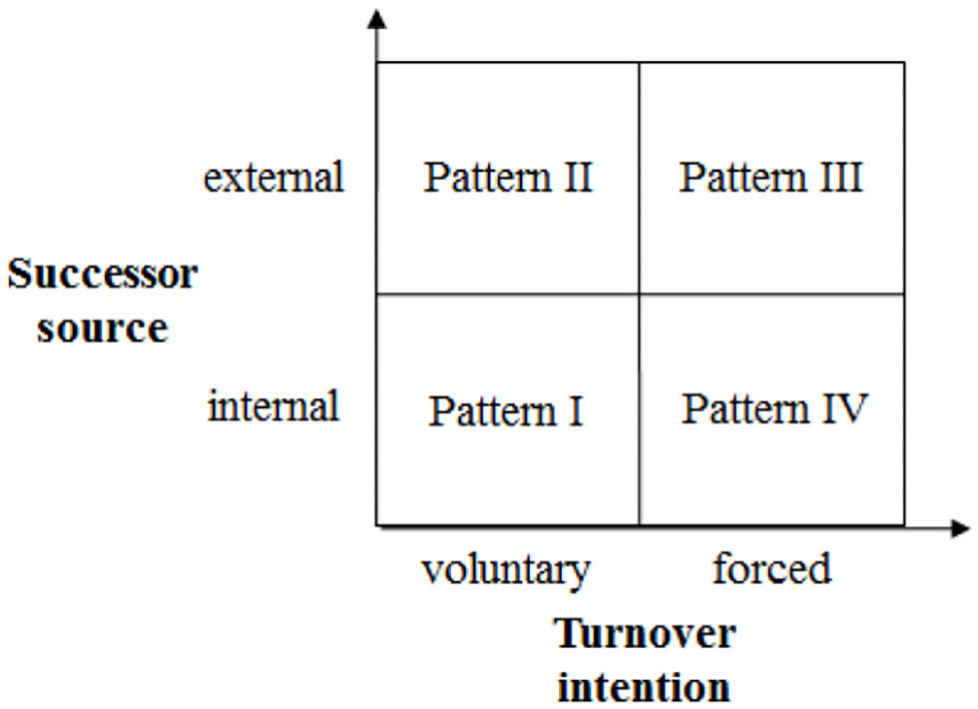
CEO turnover patterns.

According to the principal-agent theory, CEO is appointed by the board of directors in a multinational corporation, and the board of directors has the power to dismiss a CEO and employ a new one any time (Owen and Temesvary, 2019). Therefore, as far as the willingness to leave is concerned, CEO turnover in a cross-cultural context can be divided into two types: the first one is voluntary and the next is forced. Voluntary resignation is the voluntary behavior of CEO in a multinational corporation who voluntarily abandons his position and transfers the power to the successor. For example, when the tenure expires, CEO does not seek to continue to serve as a CEO but chooses to resign and give up his position voluntarily; CEO leaves office in a planned way, etc. Forced resignation is the passive act of CEO in a multinational corporation, and CEO is forced to abandon his position and transfer the power to the successor. For example, the board of directors terminates the tenure of CEO forcibly because of his legal liability; CEO is forced to resign under various pressures, etc. Whatever the reasons of CEO for departure are, the multinational corporation must find a successor as soon as possible. As for the sources of succession, the successor can come from the internal of the multinational corporation on the one hand, which is to employ an existing employee as a CEO; on the other hand, from the external of the multinational corporation, which is to employ a CEO outside the enterprise. To sum up, CEO turnover patterns are shown in Figure 1. In addition, according to the social political model of CEO turnover (Fredrickson et al., 1988), the successor, especially for the one coming from the external of the enterprise, must face the pressure and power struggle of the original TMT members (Karaevli, 2007). Therefore, the successor must prove his ability and establish his prestige in the quickest and most powerful way to eliminate the pressure of the political conflict and power struggle within the team in a cross-cultural context (Liu, 2015).

### Team Creativity

Team creativity is a collection of new and useful ideas, and it is an innovative result formed by team members through collective decision-making after fully considering various views (Dong et al., 2017). In the fierce international market competition, team creativity is an important source for the multinational corporation to carry out the innovative practice and maintain the competitive advantage; improving team creativity has become the key for a multinational corporation to develop (Lin and Yi, 2020). Ma et al. (2020) found if the team leader could identify the knowledge needed for the innovation and motivate team members with the different knowledge; it would help to improve the level of team creativity. Therefore, scholars have studied what kind of leadership style of the team leader can stimulate team creativity more effectively (Zhao et al., 2020). CEO turnover means replacing the team leader with a different leadership style for TMT so as to investigate whether the successor can properly handle the various problems and stimulate TMT creativity more effectively. Thus, it can be seen that this study is an extension and expansion of the existing research on team creativity.

### Cross-Cultural Context

When a multinational corporation enters the international market, it will inevitably face all kinds of cross-cultural differences. The key to the successful operation of a multinational corporation is to correctly understand and actively deal with these cross-cultural differences. Cross-cultural management is to overcome the differences between the heterogeneous cultures, reshape the unique culture of the enterprise, and, finally, create the management behavior with outstanding performance (Sun, 2008). TMT members of a multinational corporation often have the cross-cultural context, which makes it easy to have a variety of cognitive conflicts among TMT members (Rong et al., 2019b). However, the multicultural background can also provide diversified information and more alternatives for the collective decision-making of TMT (Pisani et al., 2018). Therefore, the focus of this study is whether the successor can grasp the positive role brought by the cross-cultural context of TMT in a multinational corporation and avoid its negative impact after the CEO turnover.

## Hypotheses Development

### The Impact of CEO Turnover on TMT Creativity in a Cross-Cultural Context

How the leadership behavior affects team creativity is a widely debated topic, and there is no consensus yet. According to the theory of creativity composition, creativity is closely related to knowledge, intelligence (including intellect, observation, memory, and other abilities), and good personality; they interact and influence one another, and ultimately determine the creativity level of a person, and the leadership behavior guides team members to form the good personalities by changing their knowledge and intelligence so as to change their intrinsic motivations and improve their creativity levels (Chen et al., 2013). Zhao et al. (2019) found that CEO-empowering leadership endows TMT members with greater management authority through authorization so that they have the opportunities to engage in a work different from the past, acquire the new knowledge and experience, and improve their abilities so as to generate the diversified cognition and improve their creativities in teamwork; Chen et al. (2016) found that CEO transformational leadership can stimulate the strong learning motivations of TMT members by carrying out positive management changes and lead TMT to conduct the team learning and creative thinking to acquire new knowledge and innovative skills so as to effectively enhance TMT creativity. All of these show that the different CEOs will take different ways to stimulate TMT creativity. And, next, this study will analyze one by one how the internal or external successor stimulates TMT creativity in combination with the different CEO turnover patterns under the cross-cultural background.

As shown in Figure 1, Pattern I represents CEO voluntary resignation/internal succession, in which the former CEO voluntarily abandons his post, and the board of directors of the multinational corporation appoints a successor coming from the internal of the enterprise. In this pattern, the CEO voluntarily gives up the position due to the expiration of his tenure or due to other reasons so as to avoid aggravating the internal contradictions of TMT and naturally resolve the possible interpersonal conflicts within TMT (Rong et al., 2019b). At the same time, the successor coming from the internal of the enterprise is usually familiar with the corporate culture formed by the multinational corporation and the existing operation mechanism of TMT, and understands the team tasks, objectives, and operation rules so he can quickly adapt to the new job requirements and lead TMT members to continue to complete the team mission (Shao and Zhu, 2017). And the successor coming from the enterprise is also very clear about the current situation and problems faced by TMT, so it is easier for him to put forward some new solutions to integrate TMT (such as changing the existing structure of TMT through the personnel changes; encouraging TMT members to participate more actively in the strategic decision-making and share their personal views, etc.) for improving team operation based on the former CEO, take the new measures (for example, the internal successor can promote the frank communications and exchanges among TMT members by cultivating a good team working atmosphere and strengthen the cooperation among TMT members) to resolve the various contradictions and problems in the decision-making process of TMT, and stimulate TMT creativity. For example, the internal successor can choose to follow the effective ways of the former CEO to stimulate TMT creativity, advocate a more open and inclusive team working atmosphere than before, and encourage TMT members with the different knowledge and background to share their opinions more actively and communicate with one another frankly so as to obtain the more creative strategies. Therefore, Hypothesis 1 is proposed as follows:

H1: the CEO voluntary resignation/internal succession pattern has a significant positive impact on TMT creativity in a cross-cultural context.

Pattern II represents CEO voluntary resignation/external succession, in which the former CEO voluntarily abandons his post and the board of directors of the multinational corporation appoints a successor coming from the external of the enterprise. In this pattern, the CEO voluntarily gives up the position, which ensures the stability of the internal structure for TMT. However, due to the lack of a suitable successor inside, the enterprise has to choose and employ a successor from the external of the enterprise. And the new external successor usually faces the following problems in a cross-cultural context (Shao and Zhu, 2017). First, the external successor needs to integrate into the new team, strive to become a member of TMT, and get the trust and support from the other team members as soon as possible. Second, the external successor needs to properly handle the various contradictions within the team so that TMT can get rid of the negative impact that is caused by the former CEO resignation or other reasons as soon as possible and lead TMT to complete the new team mission. Although facing many difficulties, the external successor will also bring new management concepts and methods to TMT, such as making the new operation rules in a cross-cultural context, standardizing the operation process of TMT, or redefining the team objectives, and making more diversified action plans based on the different perspectives generated by the multicultural conflicts to ensure the team tasks are completed (Liu et al., 2019). In addition, the external successor can also input a lot of new knowledge for TMT, help TMT members broaden their knowledge boundaries, and enrich the existing knowledge systems so as to improve their creativities (Ali et al., 2019). For example, in order to ensure the effective implementation of the new strategies, the external successor often implants the new management concepts into TMT, actively shares the personal knowledge with TMT members, and imparts his successful experience so as to enrich the existing knowledge system of TMT members, and improve both their cognitive level and innovation decision-making ability. Therefore, Hypothesis 2 is proposed as follows:

H2: CEO voluntary resignation/external succession pattern has a significant positive impact on TMT creativity in a cross-cultural context.

Pattern III represents CEO forced resignation/external succession, in which the former CEO is forced to abandon his post and the board of directors of the multinational corporation appoints a successor coming from the external of the enterprise. In this pattern, the CEO has to give up the position because of his legal liability or the other reasons, and, due to the lack of the suitable candidate, the multinational corporation can only choose a suitable successor from the external of the enterprise. However, the abnormal departure of the former CEO often means there are many fierce contradictions and conflicts within TMT, or greater challenges have to be faced by TMT (Liu et al., 2013). Therefore, the new external successor who joins the team needs to spend a lot of time and energy to solve the problems of TMT itself at first, for example, dealing with the unfinished tasks handed down by the former CEO, straightening out the complex interpersonal relationship caused by the multicultural conflict among TMT members, and so on so that the team can get rid of the difficulties as soon as possible and resume the normal operation (Liu et al., 2019). In this case, the external successor not only cannot give full play to his management ability and stimulate TMT creativity but also may fall into the complicated interpersonal conflicts within the team and inhibit the formation of TMT creativity. For example, although the external successor can bring the new management ideas, knowledge, and methods to TMT, when the former CEO is forced to leave, TMT members tend to pay more attention to the existing contradictions, conflicts, and interest disputes within the team rather than the measures of management change taken by the external successor and whether the measures can enhance TMT creativity. Therefore, Hypothesis 3 is proposed as follows:

H3: The CEO forced resignation/external succession pattern has a significant negative impact on TMT creativity in a cross-cultural context.

Pattern IV represents CEO forced resignation/internal succession, in which the former CEO is forced to abandon his post and the board of directors of the multinational corporation appoints a successor coming from the internal of the enterprise. In this pattern, the CEO has to give up his position; it makes TMT face a lot of difficulties as mentioned above, and fall into an awkward situation of no leader (Liu et al., 2013). In this case, the successor coming from the multinational corporation is often familiar with the problems faced by TMT, such as the interpersonal conflicts, the unbalanced distribution of the interests, so he will take some policies and measures different from the former CEO to try to solve the various cultural conflicts within the team, balance the various interests, and try his best to mobilize the enthusiasm and the creativity of TMT members so that the team can return to the normal operation as soon as possible (Shao and Zhu, 2017). For the internal successor, it requires him to have excellent management abilities and skills, and be good at dealing with the conflicts caused by the diversity of the team culture. Of course, if the internal successor can meet these requirements, it will help to promote the creative thinking ability of TMT members and improve the overall level of TMT creativity. For example, the smart internal successor will always remind himself to take more effective measures to resolve the various conflicts within the team, enhance the cohesion and centripetal force of TMT, and stimulate TMT creativity through collective wisdom. Therefore, Hypothesis 4 is proposed as follows:

H4: The CEO forced resignation/internal succession pattern has a significant positive impact on TMT creativity in a cross-cultural context.

Through the above analysis, it is not difficult to find that, in the case of CEO turnover in the multinational corporations, the internal successor mainly integrates the composition and structure of the existing TMT to achieve a more reasonable allocation of human resources within the team so as to continuously cultivate the cohesion and centripetal force of TMT, stimulate the innovation vitalities of TMT members, and enhance TMT creativity; while the external successor mainly inputs the new ideas and knowledge to TMT, implements the positive management changes within the multinational corporation, and leads TMT members to participate in the enterprise innovation practice so as to improve their innovation abilities. However, compared with the CEO's voluntary resignation, when the CEO is forced to leave, both the internal successor and the external successor need to face the more complex interpersonal relationships, and properly handle and solve the existing conflicts and interest disputes within TMT.

### The Moderating Effect of Leadership Identity

Leadership identity is a state in which subordinates define themselves according to their relationship with a leader (Xu and Wang, 2016). Li and Sun (2015) found subordinates with high leadership identity have stronger imitation and learning willingness to leadership attitude and behavior. This is because subordinates with high leadership identity tend to internalize the interests, goals, and values of leaders, and are even willing to change their self-concept to make their values, beliefs, and behaviors more similar to those of the leaders. In addition, subordinates with high leadership identity are often highly sensitive to the expectations of the leaders and meet the expectations through actions (Aron, 2003). For example, when leaders show the authoritarian behavior, the team with high leadership identity will show such behavior more, which means leadership identity will strengthen the learning and imitation to leaders of subordinates (Li and Sun, 2015).

Based on the above analysis, this study believes whether the successor can stimulate TMT creativity that depends on whether TMT members have a high sense of identity with him when the CEO changes in a cross-cultural context, that is, the higher the sense of identity with the successor, the more likely TMT members are to be highly sensitive to the expectations of the successor and to strengthen the imitation and learning of the innovative ideas and behaviors of the successor, and try to be consistent with the leader so as to stimulate TMT creativity (Ambrose et al., 2018). On the contrary, if leadership identity is low, TMT members are more inclined to work in their own ways rather than learning the innovation ideas and behaviors of the successor, so it is difficult to improve the overall creativity level for TMT (Kim, 2017). It can be seen that leadership identity has a moderating effect between CEO turnover and TMT creativity. Specifically, in the pattern of CEO voluntary resignation/internal succession, after the former CEO leaves voluntarily, the reason why the internal successor can succeed is often because of his high prestige and recognition coming from TMT members so that he can stand out and become a new CEO (Shao and Zhu, 2017). This high recognition will also enable the successor to obtain relatively long-lasting support and trust, which make the innovation concept and innovation strategy proposed by the successor be easy to be implemented. In the pattern of CEO voluntary resignation/external succession, although the CEO voluntary resignation ensures the stability of the internal structure for TMT, the successor coming from the external must obtain the leadership identity of TMT members in order to carry out the work smoothly, and the higher the sense of identity, the more stable the leadership position of the external successor within TMT, which is more conducive to stimulate TMT creativity by the external successor (Shao and Zhu, 2017; Huang et al., 2019). In the pattern of CEO forced resignation/external succession, the CEO forced resignation often means the internal conflicts of TMT become more and more serious, which makes it more difficult for the external successor to obtain the leadership identity of TMT members. Of course, if the external successor can solve the internal conflicts of the team as soon as possible and improve the leadership identity of TMT members, it will be of great help to enhance team cohesion and creativity. In the pattern of CEO forced resignation/internal succession, after the former CEO is forced to leave office, TMT urgently needs to get rid of the trouble of the fierce conflicts as soon as possible and move into the normal operation, and the successor coming from the internal of the enterprise is often expected to be high, so he has a high sense of leadership identity, which enables team members to move closer to the internal successor spontaneously and learn his innovative ideas and improve the creativity themselves (Shao and Zhu, 2017). Therefore, Hypothesis 5 is proposed as follows:

H5: In a cross-cultural context, leadership identity has a regulatory effect between CEO turnover and TMT creativity.
H5a: In a cross-cultural context, leadership identity can positively regulate the impact of CEO voluntary resignation/internal succession pattern on TMT creativity.
H5b: In a cross-cultural context, leadership identity can positively regulate the impact of CEO voluntary resignation/external succession pattern on TMT creativity.
H5c: In a cross-cultural context, leadership identity can negatively regulate the impact of CEO forced resignation/external succession pattern on TMT creativity.
H5d: In a cross-cultural context, leadership identity can positively regulate the impact of CEO forced resignation/internal succession pattern on TMT creativity.

**Figure 2:**
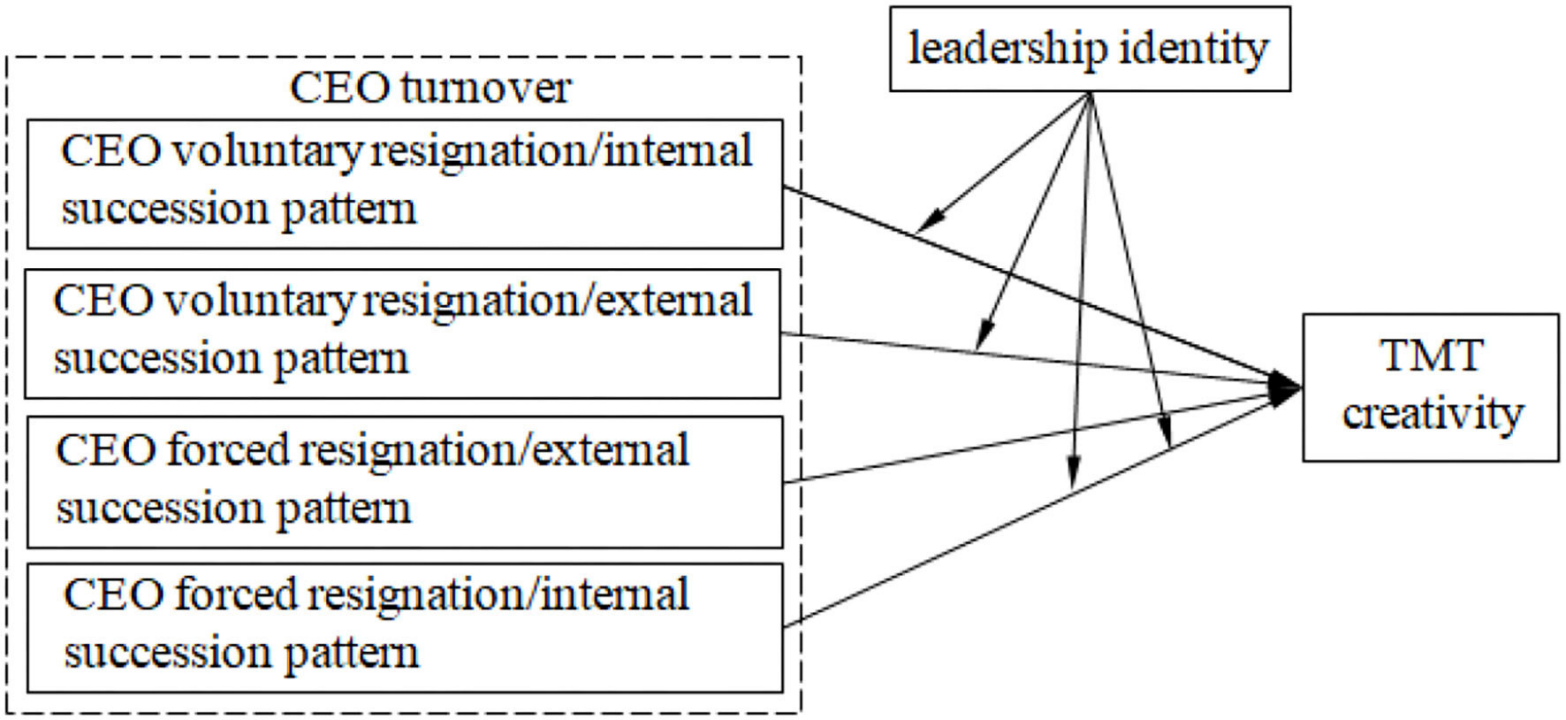
The theoretical model.

To sum up, the theoretical model of this study is shown in Figure 2.

## Method

### Participants, Procedure, and Materials

Participants were members of TMTs in all kinds of multinational corporations in Zhejiang Province, Jiangsu Province, and Shanghai City in China. A survey with people who were studying for MBA or EMBA at universities in Shanghai City was conducted, and who were also executive staff in the multinational corporations that had changed CEO in the past 5 years. Simultaneously, the people who were their colleagues in the TMT in the same corporation were also invited to fill out the survey forms with the help of the student participants, and then, all of the completed survey forms were collected.

This study was approved by the Research Ethics Committee of Shanghai Normal University, and all the participants provided the written informed consent prior to taking part in the study. And then, data used in this study were collected by anonymous questionnaires, which were designed according to the existing mature scales. Before the formal investigation, in order to ensure the accuracy of the questionnaire, two senior experts in the field of human resource management were invited to check the contents of the survey items carefully, and a small-scale questionnaire survey with only eight TMTs was conducted. After these, the semantic expression of some items in the questionnaire was modified so as to make them easier for the participants to understand than before. Finally, in order to reduce the common methodological bias caused by the same participants or the data sources, the order of the measurement items was disrupted to reduce the self-defense consciousness of the participants, and the revised questionnaire (see Appendix for details) was used to carry out the formal survey.

About 127 TMTs were surveyed; 1,132 survey forms were distributed, and 954 were collected (84.28% response rate), of which 903 valid forms were obtained after eliminating the forms with the invalid responses, such as those with missing responses or with errors in responses (response rate = 79.77%), the participants (40.64% women) were comprised members of 104 TMTs, and each team consisted of nine members (*SD* = 3.56, range 6–13) on average. Among them, CEO voluntary resignation/internal succession pattern includes 32 TMTs (276 persons), CEO voluntary resignation/external succession pattern includes 27 TMTs (252 persons), the CEO forced resignation/external succession pattern includes 21 TMTs (192 persons), the CEO forced resignation/internal succession pattern includes 24 TMTs (183 persons). The descriptive statistics of the samples are shown in Table 1.

**Table 1:** The descriptive statistics of the samples.

**Demographic characteristics**	**Characteristic range**	**CEO voluntary resignation/internal succession**		**CEO voluntary resignation/external succession**		**CEO forced resignation/external succession**		**CEO forced resignation/internal succession**	
Gender	Male	145	16.1%	139	15.5%	119	13.2%	97	10.8%
	Female	127	14.1%	113	12.6%	73	8.1%	86	9.6%
Age	30–40	56	6.2%	43	4.8%	47	5.2%	35	3.9%
	41–50	104	11.6%	87	9.7%	78	8.7%	72	8.0%
	>50	112	12.4%	122	13.6%	67	7.4%	76	8.5%
Education level	Junior college or below	41	4.6%	47	5.2%	32	3.6%	38	4.2%
	Bachelor	134	14.9%	98	10.9%	83	9.2%	69	7.7%
	Master or post-graduate	97	10.8%	107	11.9%	77	8.6%	76	8.4%
Tenure	<3 years	22	2.5%	16	1.8%	23	2.6%	13	1.5%
	3–5 years	46	5.1%	48	5.3%	31	3.5%	51	5.7%
	6–10 years	92	10.2%	105	11.6%	76	8.4%	42	4.7%
	>10 years	112	12.4%	83	9.2%	62	6.9%	77	8.6%

### Measures

The main variables were CEO turnover, leadership identity, and TMT creativity. Because some multinational corporations did not have a CEO, but through the establishment of a president or general manager to be responsible for the implementation of the corporate decisions and the management of the daily affairs, CEO turnover in this study referred to the following: (1) in a company with a CEO, whether the CEO change was the standard; (2) in a company without a CEO, whether the president or general manager change was the standard. In this study, a questionnaire survey was used to understand the CEO turnover patterns of the surveyed enterprises, making CEO voluntary resignation/internal succession pattern = 1, CEO voluntary resignation/external succession pattern = 2, CEO forced resignation/external succession pattern = 3, CEO forced resignation/internal succession pattern = 4. To ensure reliability and validity, the survey was designed for the variables of leadership identity and TMT creativity to be assessed, using a scale that had been widely used in both China and countries like Britain and North America, and that was scored on a Likert five-point scale, with 1 = strongly disagree to 5 = strongly agree as anchors.

#### Leadership Identity

The leadership identity scale used by Chen and Chen (2017) was modified and adopted, which included seven items, a sample of which was “I'm happy for the success of the CEO successor.” After data detection, it was found the Cronbach's α (0.84) of the leadership identity scale was >0.7, which indicated the internal consistency of the scale was good and the reliability was high. The square root (0.79) of the mean variance for the variable was >0.70, which indicated the scale had good convergence validity; the CFA analysis of leadership identity found the normalized factor loads of the seven items were >0.6 (*p* < 0.001), and the overall fit of leadership identity model was good (χ^2^/*df* = 2.06, *p* < 0.001, RMR = 0.06, GFI = 0.94, IFI = 0.91, CFI = 0.97, RMSEA = 0.07), which indicated the leadership identity items represented the different constructs and had the discriminant validity. Generally, the leadership identity scale had good reliability and validity.

#### TMT Creativity

The TMT creativity scale used by Shin and Zhou (2007) was adopted, which included four items, a sample of which was “TMT members often produce the new suggestions and new ideas.” After data detection, it was found the Cronbach's α (0.86) of the TMT creativity scale was >0.7, which indicated the internal consistency of the scale was good and the reliability was high. The square root (0.83) of the mean variance for the variable was >0.70, which indicated the scale had good convergence validity; the CFA analysis of TMT creativity found the normalized factor loads of the four items were >0.6 (*p* < 0.001), and the overall fit of TMT creativity model was good (χ^2^/*df* = 2.13, *p* < 0.001, RMR = 0.05, GFI = 0.91, IFI = 0.95, CFI = 0.93, RMSEA = 0.06), which indicated the TMT creativity items represented the different constructs and had the discriminant validity. Generally, the TMT creativity scale had good reliability and validity.

#### Control Variables

Findings in a previous study had shown the gender, age, tenure, and education level of TMT members affected the operation processes of TMT (Zhu and Peng, 2017). Accordingly, these demographic characteristics were taken as the control variables, and relevant data were obtained for them in the survey items.

### Common Method Deviation Test

This study used the Harman single factor test to conduct the exploratory factor analysis, six factors were selected to explain 77.23% of the total variance, and the first factor explained 26.81%. Thus, it could be seen that there was no serious common methodological bias in this study.

### Data Aggregation Analysis

To aggregate the individual variable data of TMT members to the team level, the R_wg_ index was adopted to evaluate the consistency within the group of leadership identity and TMT creativity, and the intraclass correlation coefficient (ICC) indexes (1) and (2) were used to estimate the heterogeneity between groups. The results showed R_wg_ index medians of leadership identity and TMT creativity were 0.95 (*M* = 0.92) and 0.93 (*M* = 0.91), all of which showed the evaluations of group members were consistent for each variable. The values of ICC (1) on leadership identity and TMT creativity were 0.24 and 0.21, and the values of ICC (2) were 0.65 and 0.63. Therefore, the individual variable data of leadership identity and TMT creativity could be aggregated to the team level.

## Results

The multiple regressions are performed to analyze the impact of CEO turnover on TMT creativity and the moderating effect of leadership identity between CEO turnover and TMT creativity. The data for the variables are centralized before the multiple regression analysis so as to further eliminate the multiple collinearities among the variables.

### Descriptive Analysis

Table 2 shows the descriptive statistical results and correlations between the main variables.

**Table 2:** The descriptive statistics and correlation coefficients for the variables.

	**1**	**2**	**3**	**4**	**5**	**6**	**7**	**8**	**9**	**10**
1. Gender										
2. Age	0.04									
3. Tenure	0.02	0.06								
4. Education level	−0.02	−0.03	−0.03							
5. Pattern I	0.11	0.02	0.05	0.08						
6. Pattern II	0.06	0.07	−0.08	0.03	0.06					
7. Pattern III	0.09	0.10	−0.10	0.04	0.07	0.09				
8. Pattern IV	−0.04	0.04	0.06	0.07	0.05	0.06	0.08			
9. Leadership identity	0.10	0.07	0.17^*^	0.04	0.27^**^	0.23^**^	−0.22^*^	0.25^**^		
10. TMT creativity	0.08	−0.16^*^	−0.07	0.18^*^	0.30^**^	0.32^***^	−0.20^*^	0.28^**^	0.26^**^	
*M*	1.18	3.49	2.78	3.16	/	/	/	/	3.54	3.76
*SD*	0.36	0.72	1.05	1.09	/	/	/	/	1.22	1.19

*N = 104*.

* *p < 0.05*,

** *p < 0.01, and*

*** *p < 0.001*.

According to Table 2, Pattern I, Pattern II, and Pattern IV are all significantly positively correlated with leadership identity and TMT creativity, respectively; Pattern III is significantly negatively correlated with both leadership identity and TMT creativity; and leadership identity is significantly positively correlated with TMT creativity. Among the control variables, age is significantly negatively correlated with TMT creativity; tenure is significantly positively correlated with leadership identity, and education level is significantly positively correlated with TMT creativity.

### Hypotheses Testing

The results of the multiple regression analysis for the hypotheses are shown in Table 3.

**Table 3:** Multiple regression analysis.

**Variable**	**Dependent variable: TMT creativity**								
	**Model 1**	**Model 2**	**Model 3**	**Model 4**	**Model 5**	**Model 6**	**Model 7**	**Model 8**	**Model 9**
**Control variable**									
Gender	0.027	0.031	0.024	0.021	0.030	0.028	0.033	0.026	0.031
Age	−0.086	−0.105	−0.074	−0.097	−0.082	−0.079	−0.102	−0.088	−0.075
Tenure	−0.064	−0.049	−0.054	−0.038	−0.061	−0.046	−0.063	−0.042	−0.059
Education level	0.082	0.103	0.073	0.090	0.085	0.077	0.095	0.089	0.101
**Independent variable**									
Pattern I		0.436^**^				0.403^**^			
Pattern II			0.512^***^				0.491^***^		
Pattern III				−0.137				−0.129	
Pattern IV					0.417^**^				0.410^**^
**Mediating variable**									
Leadership identity						0.228^**^	0.265^**^	0.209^**^	0.187^**^
**Product term**									
Pattern I × Leadership identity						0.115^*^			
Pattern II × Leadership identity							0.128^*^		
Pattern III × Leadership identity								−0.056	
Pattern IV × Leadership identity	0.106^*^								
*R* ^2^	0.061	0.256	0.269	0.152	0.318	0.275	0.314	0.166	0.372
*F*	1.237	4.413^**^	4.265^**^	1.714	5.169^**^	5.124^**^	5.131^**^	1.708	6.015^**^
*ΔR* ^2^	–	0.215	0.241	0.133	0.305	0.223	0.302	0.152	0.314
*ΔF*	–	2.705^*^	2.755^*^	1.662	3.764^*^	3.196^*^	3.282^*^	1.635	4.112^**^

* *p < 0.05*,

** *p < 0.01, and*

*** *p < 0.001*.

According to Models 2, 3, and 5 in Table 3, it can be seen Pattern I, Pattern II, and Pattern IV separately have a significant positive effect on TMT creativity (*r* = 0.436, *p* < 0.01; *r* = 0.512, *p* < 0.001; *r* = 0.417, *p* < 0.01); therefore, hypotheses 1, 2, and 4 are supported. In addition, although the regression coefficient that Pattern III affects TMT creativity is negative, the effect is not significant, so Hypothesis 3 is not verified.

**Figure 3:**
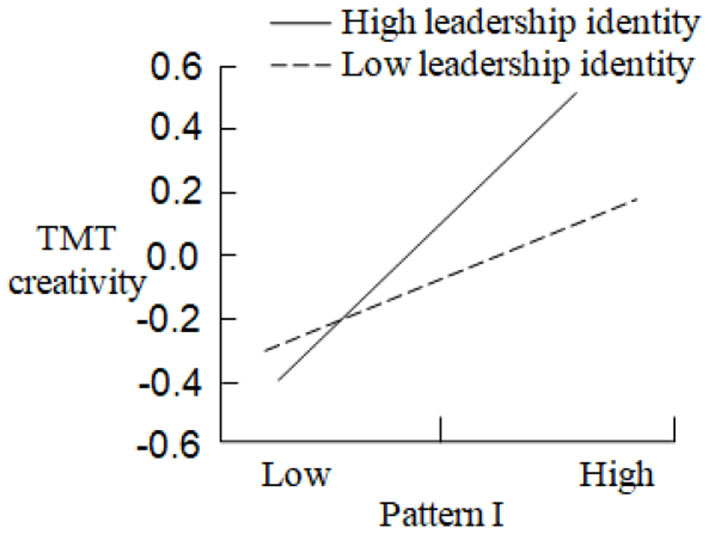
The moderating effect of leadership identity in the relationship between pattern I and TMT creativity.

**Figure 4:**
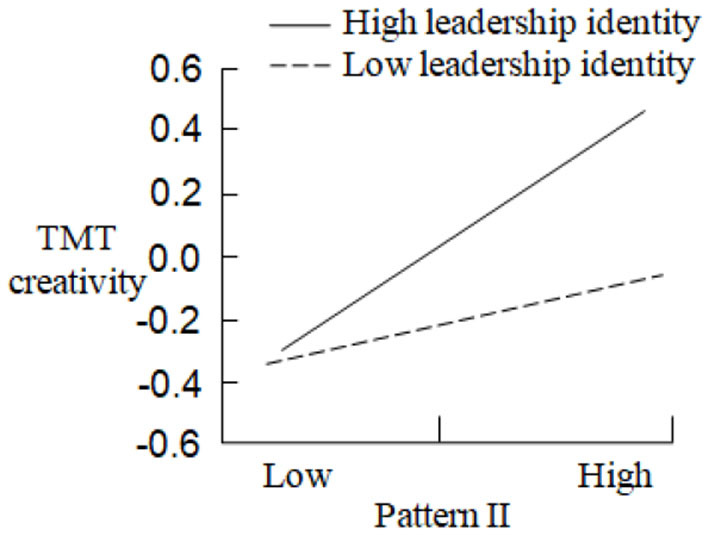
The moderating effect of leadership identity in the relationship between pattern II and TMT creativity.

**Figure 5:**
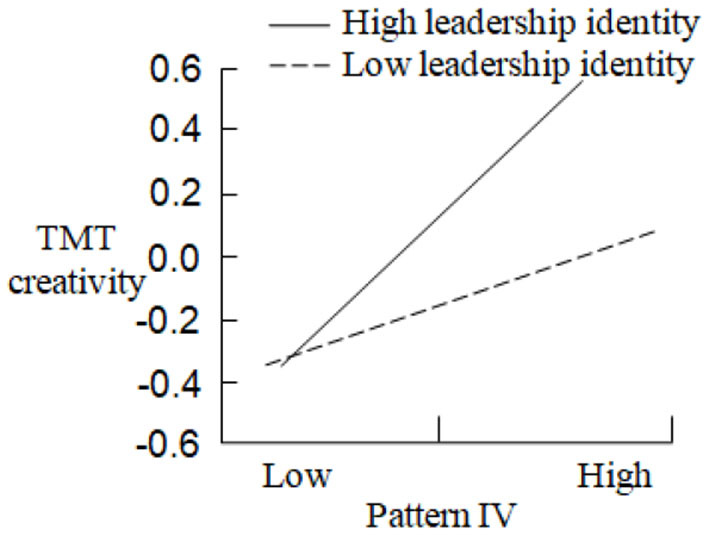
The moderating effect of leadership identity in the relationship between pattern IV and TMT creativity.

In order to test the moderating effect of leadership identity between CEO turnover and TMT creativity, Model 6, Model 7, Model 8, and Model 9, respectively, examine the regression results of the product terms between Pattern I, Pattern II, Pattern III, Pattern IV, and leadership identity on TMT creativity. Compared with Model 2, Model 3, and Model 5, Model 6 (Δ*F* = 3.196, *p* < 0.05), Model 7 (Δ*F* = 3.282, *p* < 0.01), and Model 9 (Δ*F* = 4.112, *p* < 0.05) are significantly improved, and Δ*R*^2^ is 0.223, 0.302, and 0.314, respectively. Therefore, leadership identity has the positive moderating effects between Pattern I, Pattern II, Pattern IV, and TMT creativity, and the moderating effect curves are shown in Figures 3–5: In the case of high leadership identity, Pattern I, Pattern II, and Pattern IV have greater positive impacts on TMT creativity (*Slope* = 0.563, *p* < 0.001; *Slope* = 0.489, *p* < 0.001; *Slope* = 0.502, *p* < 0.001); however, in the case of low leadership identity, Pattern I, Pattern II, and Pattern IV have less positive impacts on TMT creativity (*Slope* = 0.394, *p* < 0.001; *Slope* = 0.276, *p* < 0.001; *Slope* = 0.388, *p* < 0.001). H5a, H5b, and H5d are supported. In addition, compared with Model 4, Model 8 is not improved, and the effect is not significant, so H5c is not supported. In summary, H5 is partially supported.

## Conclusions and Discussion

### Conclusions

This study analyzed the influence of CEO turnover on TMT creativity in a cross-cultural context and explored the moderating effect of leadership identity between CEO turnover and TMT creativity. Research showed CEO voluntary resignation/internal succession pattern, CEO voluntary resignation/external succession pattern, and CEO forced resignation/internal succession pattern separately had a significant positive impact on TMT creativity in a cross-cultural context; leadership identity partially moderated the relationship between CEO turnover and TMT creativity. According to the conclusions, only CEO voluntary resignation/internal succession pattern, CEO voluntary resignation/external succession pattern, and CEO forced resignation/internal succession pattern could promote TMT creativity in a cross-cultural context, and leadership identity enhanced the positive effects of CEO voluntary resignation/internal succession pattern, CEO voluntary resignation/external succession pattern, and CEO forced resignation/internal succession pattern on TMT creativity.

### Theoretical Contributions

Unlike the previous studies on general team creativity (Lin and Li, 2020; Van Dijk et al., 2020), this study aims to explore the influence of CEO turnover on TMT creativity in a cross-cultural context and has made new contributions as follows.

First, through an in-depth analysis of the connotation about CEO turnover, this study divided CEO turnover patterns into four different types of research. As far as the existing pieces of research were concerned, the previous studies on CEO turnover had not made any distinction, which led to the different opinions on the impact of CEO turnover (Chen et al., 2019; Jarva et al., 2019). In a cross-cultural context, CEOs in the different turnover patterns were in the different situations and had different problems to solve. Therefore, according to the CEO turnover intention and the sources of successors in a cross-cultural context, this study divided CEO turnover patterns into four types: CEO voluntary resignation/internal succession, CEO voluntary resignation/external succession, CEO forced resignation/external succession, and CEO forced resignation/internal succession. Since then, the four CEO turnover patterns had been studied in depth in this study.

Second, this study explored the positive impact of CEO turnover on TMT creativity. The existing studies only focused on how leaders with different styles influenced TMT creativity from the perspective of team leaders (Chang et al., 2016; Kim, 2017), but had not studied the other antecedents of those affected TMT creativities. Based on the study of Grusky (1960), CEO turnover was introduced into the TMT research field in this study and divided into four patterns. The results showed both CEO voluntary resignation/internal succession pattern, CEO voluntary resignation/external succession pattern, and CEO forced resignation/internal succession pattern had a significant positive impact on TMT creativity in a cross-cultural context separately; it revealed the origins of TMT creativity and answered the question of whether CEO turnover could promote TMT creativity in a cross-cultural context theoretically.

Third, this study found the moderating effect of leadership identity between CEO turnover and TMT creativity in a cross-cultural context. Similar to the research of Xu and Wang (2016), this study confirmed leadership identity could regulate the influence of leaders on subordinates; the difference lied in the fact that leadership identity was introduced into the specific organizational context of CEO turnover, and whether the identification of TMT members to the successor of CEO helped to adjust the impact of CEO turnover on TMT creativity. The research on the moderating effect of leadership identity further broadened the application scope of the social identity theory and provided a new situational perspective for revealing the internal process to TMT.

### Practical Contributions

The practical contributions of this study are mainly reflected in the following aspects.

First, this study points out the directions for the enterprises in a cross-cultural context to choose the appropriate CEO turnover pattern. For the enterprises in a cross-cultural context, how to change CEO to ensure the smooth transition of the power and stimulate TMT creativity through the correct leadership of the successor is a very important issue. According to the conclusions of this study, the enterprises in a cross-cultural context may choose the most appropriate CEO turnover pattern from the following three types according to the situation of themselves; they are CEO voluntary resignation/internal succession pattern, CEO voluntary resignation/external succession pattern, and CEO forced resignation/internal succession pattern; that is because only these three CEO turnover patterns can stimulate TMT creativity and improve TMT decision-making efficiency in a cross-cultural context.

Second, this study provides theoretical guidance on how to stimulate TMT creativity through the correct leadership of the CEO in a cross-cultural context. In order to stimulate TMT creativity, based on the conclusions of this study, the enterprises in a cross-cultural context should try their best to adopt CEO voluntary resignation to reduce the harmful emotional conflicts within TMT firstly so that TMT can return to the normal operation process as soon as possible. And then, the successor should integrate into TMT as soon as he can, strive to play an exemplary role, stimulate the innovation vitality of TMT members, improve the team learning ability continuously, and realize the innovation of the decision-making through the collective wisdom.

Finally, this study makes the way for promoting the positive interaction between CEO and TMT members and realizing the harmonious coexistence between them in a cross-cultural context. The enterprises in a cross-cultural context should make full use of the moderating role of leadership identity between CEO turnover and TMT creativity, strive to create a good team-working atmosphere, cultivate the sense of leadership identity coming from TMT members to the successor so as to make the goal of team leaders and team members consistent, improve the cohesion and centripetal force of TMT, and realize the harmonious coexistence between the successor and TMT members in a cross-cultural context.

### Limitations and Directions for the Future Research

There are several limitations to this study. First, considering that there are many specific reasons for CEO turnover, it is difficult to research them one by one in a study. Therefore, this study uses the method of dividing CEO turnover types according to the willingness to leave for a CEO rather than studying CEO turnover according to the specific reasons for turnover, which may have some limitations. The future research can use the case study method to analyze the specific reasons of CEO turnover. Second, this study follows the concept and measurement of western scholars on TMT creativity, lacking the specific connotation of the variable in Chinese local environments. Especially in a cross-cultural context, the influencing factors of TMT creativity may be quite different from those in the West. Therefore, the measurement scale of TMT creativity in the context of Chinese local culture is urgently needed to be developed. Third, although this study collects the cross-sectional data through the questionnaires to verify the rationality of the theoretical model, the influence of CEO turnover on TMT creativity and the moderating effect of leadership identity are the dynamic processes, and their performance may vary at different time points. Therefore, it is necessary to adopt the longitudinal research paradigm to further reveal the intrinsic process mechanism dynamically. Fourth, this study does not consider how CEO turnover influences TMT creativity through the individual psychological responses of team members and how leadership identity plays a moderating role through an individual member. Therefore, constructing a cross-level model to analyze the effects of CEO turnover and leadership identity on individual psychological and behavioral responses of TMT members is the direction for further research.

## Data Availability Statement

The original contributions presented in the study are included in the article/supplementary material, further inquiries can be directed to the corresponding author/s.

## Ethics Statement

Written informed consent was obtained from the individual(s) for the publication of any potentially identifiable images or data included in this article.

## Author Contributions

PR was responsible for the conceptualization of the idea and formulation of the overarching research goals, as well as the methodology, data curation, formal analysis, original draft, preparation, and funding acquisition. CW verified all results and created the figures, and also assisted with writing and editing of the manuscript. PR and CW reviewed and edited drafts of the manuscript. Both authors contributed to the article and approved the submitted version.

## Conflict of Interest

The authors declare that the research was conducted in the absence of any commercial or financial relationships that could be construed as a potential conflict of interest.

## Publisher's Note

All claims expressed in this article are solely those of the authors and do not necessarily represent those of their affiliated organizations, or those of the publisher, the editors and the reviewers. Any product that may be evaluated in this article, or claim that may be made by its manufacturer, is not guaranteed or endorsed by the publisher.

## Appendix: The Questionnaire

**Control variable**

1. What is your gender? (A) male (B) female
2. How old are you? (A) 30–40 years old (B) 41–50 years old (C) >50 years old
3. What is your highest education level? (A) junior college or below (B) bachelor (C) master or post-graduate
4. How long do you serve as an executive? (A) <3 years (B) 3–5 years (C) 6–10 years (D) >10 years

**CEO turnover pattern (only for CEO)**

5. What is your pattern of succeeding as CEO? (A) CEO voluntary resignation/internal succession pattern (B) CEO voluntary resignation/external succession pattern (C) CEO forced resignation/external succession pattern (D) CEO forced resignation/internal succession pattern

**Leadership identity**

6. I am happy for the success of the CEO successor.

7. I am proud to be a subordinate to the CEO successor.
8. I trust the CEO successor completely.
9. I have great respect for the CEO successor.
10. The values that the CEO successor believes in are also important to me.
11. My values are close to those of the CEO successor.
12. The CEO successor is an example for me to learn and act.

**TMT creativity**

13. TMT members often produce the new suggestions and new ideas.
14. The new suggestions and new ideas generated by TMT members are very important.
15. The new suggestions and new ideas generated by TMT members are of great value.
16. The new suggestions and new ideas generated by TMT members contribute to the team decision-making.
